# First cross-sectional, molecular epidemiological survey of *Cryptosporidium*, *Giardia* and *Enterocytozoon* in alpaca (*Vicugna pacos*) in Australia

**DOI:** 10.1186/s13071-018-3055-6

**Published:** 2018-09-05

**Authors:** Anson V. Koehler, Mohammed H. Rashid, Yan Zhang, Jane L. Vaughan, Robin B. Gasser, Abdul Jabbar

**Affiliations:** 1 Department of Veterinary Biosciences, Melbourne Veterinary School, Faculty of Veterinary and Agricultural Sciences, The University of Melbourne, Parkville, Victoria 3010 Australia; 2 Cria Genesis, PO Box 406, Ocean Grove, Victoria 3226 Australia

**Keywords:** Alpaca (*Vicugna pacos*), Australia, *Cryptosporidium*, *Giardia duodenalis*, *Enterocytozoon bieneusi*

## Abstract

**Background:**

Eukaryotic pathogens, including *Cryptosporidium*, *Giardia* and *Enterocytozoon*, have been implicated in neonatal diarrhoea, leading to marked morbidity and mortality in the alpaca (*Vicugna pacos*) and llama (*Lama glama*) around the world. Australia has the largest population of alpacas outside of South America, but very little is known about these pathogens in alpaca populations in this country. Here, we undertook the first molecular epidemiological survey of *Cryptosporidium*, *Giardia* and *Enterocytozoon* in *V. pacos* in Australia.

**Methods:**

A cross-sectional survey of 81 herds, comprising alpacas of 6 weeks to 26 years of age, were sampled from the six Australian states (Queensland, New South Wales, Victoria, South Australia, Tasmania and Western Australia) across the four seasons. PCR-based sequencing was employed, utilising genetic markers in the small subunit of the nuclear ribosomal RNA (*SSU*) and 60-kilodalton glycoprotein (*gp60*) genes for *Cryptosporidium*, triose-phosphate isomerase (*tpi*) gene for *Giardia duodenalis* and the internal transcribed spacer region (*ITS*) for *Enterocytozoon bieneusi*.

**Results:**

PCR-based analyses of 81 faecal DNA samples representing 1421 alpaca individuals detected *Cryptosporidium*, *Giardia* and/or *Enterocytozoon* on 15 farms in New South Wales, Victoria and South Australia, equating to 18.5% of all samples/herds tested. *Cryptosporidium* was detected on three (3.7%) farms, *G. duodenalis* on six (7.4%) and *E. bieneusi* on eight (9.9%) in two or all of these three states, but not in Queensland, Tasmania or Western Australia*.* Molecular analyses of selected faecal DNA samples from individual alpacas for *Cryptosporidium*, *Giardia* and/or *Enterocytozoon* consistently showed that alpacas of ≤ 6 months of age harboured these pathogens.

**Conclusions:**

This first molecular investigation of *Cryptosporidium*, *Giardia* and *Enterocytozoon* in alpaca subpopulations in Australia has identified species and genotypes that are of likely importance as primary pathogens of alpacas, particularly young crias, and some genotypes with zoonotic potential. Although the prevalence established here in the alpaca subpopulations studied is low, the present findings suggest that crias are likely reservoirs of infections to susceptible alpacas and/or humans. Future studies should focus on investigating pre-weaned and post-weaned crias, and on exploring transmission patterns to establish what role particular genotypes play in neonatal or perinatal diarrhoea in alpacas and in zoonotic diseases in different states of Australia.

**Electronic supplementary material:**

The online version of this article (10.1186/s13071-018-3055-6) contains supplementary material, which is available to authorized users.

## Background

Internationally, alpacas (*Vicugna pacos*), the domesticated form of the South American camelid vicuña (*Vicugna vicugna*), are prized for their wool and meat [1]. In the 1860s, alpacas and llamas were imported into Australia. However, the camelid industry failed to establish at that time [2]. In the late 1980s, the modern alpaca industry began in Australia, Canada and the USA, with the importation of alpacas from South America (www.alpaca.asn.au). The Australian alpaca fibre market is worth ~ AUD 3.4 million, with an estimated total herd size of 450,000 animals [3]. Commercially farmed alpacas are usually kept in small herds (*n* ≥ 50), although farms with as many as 5000 animals exist (J. L. Vaughan, unpublished data). Maintaining the health of these herds is of utmost importance to the alpaca industry.

In addition to viruses, bacteria and parasitic helminth infections [4–6], eukaryotic microbial pathogens of alpaca, including species of *Cryptosporidium*, *Giardia*, *Eimeria* and *Enterocytozoon*, have been implicated in or inferred to cause neonatal diarrhoea [4, 7], leading to severe morbidity and mortality [4, 8–12]. Co-infections of *Cryptosporidium* and *Giardia* with other pathogens, including viruses, bacteria and other protists, such as *Eimeria* are common [4, 9, 11–15]; such co-infections are recognised to increase the severity and duration of diarrhoea [4, 11]. Young alpacas, or crias, are particularly susceptible to viral and microbial infections, with much infectious disease research being focussed on this age group [9–11, 13, 16, 17] and few studies involving older animals [17–19]. Pathogens, such as *Cryptosporidium*, *Giardia* and *Enterocytozoon*, have the potential to utilise a wide range of hosts, such as humans, wild and domestic animals, as reservoirs for zoonotic transmission [20, 21].

The accurate detection and characterisation of eukaryotic microbes is central to determining their potential infection sources and transmission routes, particularly given that there are at least 37 described species of *Cryptosporidium* [22–24], eight assemblages of *Giardia duodenalis* [25] and more than 200 genotypes of *E. bieneusi* [20] to discern. Therefore, the use of molecular (particularly PCR-based) methods has become crucial for any molecular epidemiological investigation [26]. As nothing is currently known about the diversity of such microbes in Australian alpaca herds, the aim here was to undertake the first molecular survey of *Cryptosporidium*, *Giardia* and *Enterocytozoon* in subpopulations of alpacas from 81 farms in six states of Australia.

## Methods

### Collection of faecal samples

**Fig. 1:**
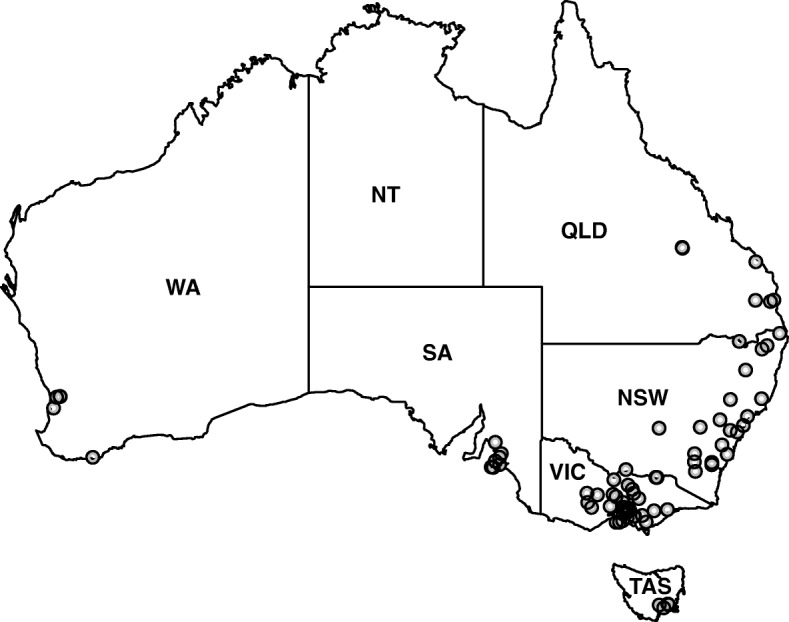
Map of Australia showing the locations of alpaca farms/herds studied. Each circle represents one alpaca farm. *QLD* Queensland , *NSW* New South Wales , *VIC* Victoria , *TAS* Tasmania, *SA* South Australia and *WA* Western Australia

Animal ethics approval (AEC no. 1413412.1) was granted by the University of Melbourne to collect faecal samples from Huacaya alpacas of 6 weeks to 26 years of age (mean: 4.8 years; both sexes) from farms in six states of Australia (Fig. 1). In total, 1421 faecal samples were collected rectally from individual alpacas from 81 herds/farms located in Queensland (QLD; *n* = 113), New South Wales (NSW; *n* = 473), Victoria (VIC; *n* = 563), Tasmania (TAS; *n* = 89), South Australia (SA; *n* = 117) and Western Australia (WA; *n* = 66) (Fig. 1). Each herd was sampled on one occasion between January 2016 and July 2017. The 81 herds comprised 9906 animals [mean herd size ± standard error of the mean (SE), was 122 ± 348; range: 13–3000]. The numbers of faecal samples from individual alpacas in each of the 81 herds varied, depending on the number of samples submitted by farmers (mean: 17.5 ± 4.8; range: 5–35). For 59 (72.9%) of the herds studied, at least 20% of the total herd size was sampled (Additional file 1: Table S1), and the average percentage of each herd sampled was 41.7% (range: 0.3–100%). Of the 1421 individual faecal samples collected, 256 were from crias (< 12 months of age), with an average herd comprising 19.9% crias; most samples were collected in the winter months (40.7%), followed by autumn (28.4%), spring (24.7%) and summer (6.2%) (Table 1).

**Table 1:** The numbers of herds (representing 1421 individual alpacas) sampled from 81 farms from six states in Australia (Fig. 1), and the numbers of pooled faecal samples that were test-positive for *Cryptosporidium* sp., *Giardia duodenalis* and *Enterocytozoon bieneusi* using specific PCR-based sequencing tools (top). Molecular results are also presented according to season in which faecal samples were collected (bottom)

	Alpacas sampled		Pathogens identified by PCR-based sequencing		
	No. of herds	No. of individuals	*Cryptosporidium* sp.	*G. duodenalis*	*E. bieneusi*
State					
NSW	26	473	1	4	3
QLD	7	113	0	0	0
SA	7	117	0	1	2
TAS	4	89	0	0	0
VIC	32	563	2	1	3
WA	5	66	0	0	0
Total	81	1421	3	6	8
Season					
Spring	23	382	0	3	3
Summer	20	369	0	0	0
Autumn	5	89	0	1	0
Winter	33	581	3	2	5
Total	81	1421	3	6	8

*Abbreviations*: *NSW* New South Wales, *QLD* Queensland, *SA* South Australia, *TAS* Tasmania, *VIC* Victoria, *WA* Western Australia

### Genomic DNA isolation and molecular analyses

Faecal samples collected from individual alpacas (*n* = 5 to 35, in most cases) from each of the 81 herds/farms (Additional file 1: Table S1) were subjected to sucrose flotation (Methods 3.1 and 3.2 in [27]). During this procedure, ~ 20% of the final suspensions (step 4 of Method 3.2 in [27]) derived from all individual faecal samples from each of the herds were pooled, resulting in 81 ‘pooled faecal concentrates’ representing the individual farms. Genomic DNA was isolated from 200 μl each of these concentrates using Method 3.3 [27]. This latter method has been shown to eliminate any faecal constituents that might be inhibitory to PCR [28]. As our goal was to investigate the presence of *Cryptosporidium*, *Giardia* and *Enterocytozoon* populations on the farms, and, where possible, to identify respective species, genotypes or assemblages, we thawed all 81 purified faecal genomic DNA samples (same codes/designations as farms) and subjected them to nested PCR-based sequencing. Subsequently, as required, DNA samples from *individual* faecal samples (represented in the pooled samples) were prepared [27] and subjected to the same PCR-based analyses.

Established nested PCRs were conducted utilising regions in the small subunit of nuclear ribosomal RNA (*SSU*), the 60-kilodalton glycoprotein (*gp60*) gene (for *Cryptosporidium*; [29]) and the triose-phosphate isomerase (*tpi*) genes, (for *G. duodenalis*; [29]) as well as the internal transcribed spacer (*ITS*) of nuclear ribosomal DNA (for *E. bieneusi*; [30]). For each assay, known test-positive, test-negative and no-template controls were included in every round of every PCR run. No-template (negative) controls were included at all steps, and no-template controls were ‘carried over’ from the primary to the secondary (nested) PCR. Following PCR, amplicons were examined on standard ethidium bromide-stained 1.5% agarose gels using TBE (65 mM Tris-HCl, 27 mM boric acid, 1 mM EDTA, pH 9; Bio-Rad, Hercules, CA, USA) as the buffer and a 100 bp-DNA ladder (Promega, Madison, WI, USA) as a size marker. Aliquots of individual amplicons were treated with ExoSAP-IT (Affymetrix, Santa Clara, CA, USA) and directly sequenced in both directions (BigDye Terminator v.3.1 chemistry, Applied Biosystems, Foster City, CA, USA) using the same primers employed in the (respective) secondary PCR. Forward and reverse sequences were visually inspected, assembled using the program Geneious v.11.1.2 [31] and compared with other sequences in the GenBank database (NCBI) using the blastn program. Sequences were deposited in the GenBank database under  accession numbers MH341585-MH341587 (*SSU*), MH346121 and MH346122 (*gp60*), MH346123 and MH346124 (*tpi*), and MH342036-MH342038 (*ITS*).

## Results

### *Cryptosporidium*

*Cryptosporidium* was detected in three of all 81 samples tested (Table 2):

**Table 2:** Summary of all pathogen species, genotypes and/or assemblages identified in alpaca herds from 81 farms from six states in Australia (Fig. 1) based on PCR-based sequencing of particular genetic markers. The GenBank accession numbers of respective sequences are listed

Pathogen identified	Farm/herd/sample code	Genetic marker used	Pathogen species/genotype/assemblage identified by PCR-based sequencing	GenBank accession no.
*Cryptosporidium* sp.	CsNSW26	*SSU*	*Cryptosporidium ubiquitum*	MH341585
	CsVIC15^a^	*SSU*	*C. parvum*	MH341586
		*gp60*	*C. parvum* IIaA20G3R1	MH346121
	CsVIC25^a^	*SSU*	*C. cuniculus*	MH341587
		*gp60*	*C. cuniculus* VbA25	MH346122
*Giardia duodenalis*	CsNSW7	*tpi*	*Giardia duodenalis* AI	MH346123
	CsNSW9	*tpi*	*G. duodenalis* AI	MH346123
	CsNSW11	*tpi*	*G. duodenalis* E	MH346124
	CsNSW21	*tpi*	*G. duodenalis* AI	MH346123
	CsSA3	*tpi*	*G. duodenalis* AI	MH346123
	CsVIC27	*tpi*	*G. duodenalis* AI	MH346123
*Enterocytozoon bieneusi*	CsNSW6	*ITS*	*Enterocytozoon bieneusi* genotype ALP1	MH342036
	CsNSW11	*ITS*	*E. bieneusi* genotype ALP3	MH342037
	CsNSW20^a^	*ITS*	*E. bieneusi* genotype P	MH342038
	CsVIC16	*ITS*	*E. bieneusi* genotype ALP1	MH342036
	CsVIC22	*ITS*	*E. bieneusi* genotype ALP1	MH342036
	CsVIC23	*ITS*	*E. bieneusi* genotype P	MH342038
	CsSA3	*ITS*	*E. bieneusi* genotype ALP1	MH342036
	CsSA7	*ITS*	*E. bieneusi* genotype ALP1	MH342036

^a^Reported from a cria of ≤ 6 months of age

(i) *C. ubiquitum* was detected in sample CsNSW26. The *SSU* sequence (814 bp) (GenBank: MH341585) from this sample was identical to the sequence with accession no. JN812216 [17] representing *C. ubiquitum* from an alpaca from Peru and 52 other sequences originating from humans, other animals or environmental samples.

(ii) *C. parvum* was detected in the sample from farm CsVIC15. The *SSU* sequence obtained (785 bp) (GenBank: MH341586) was identical to that with accession no. MF074664 and more than 100 other *C. parvum* sequences (in the GenBank database) representing human and other animal hosts. The electropherogram revealed multiple peaks at 7 nucleotide sites, suggesting a mixed infection. When samples from individual alpacas (*n* = 13) from CsVIC15 were individually tested, *C. parvum* IIaA20G3R1 was identified; the *gp60* sequence (309 bp) (GenBank: MH346121) was identical to that of *C. parvum* IIaA20G3R1 (GenBank: JF727804; [32]).

(iii) *C. cuniculus* was detected in the sample from farm CsVIC25; the *SSU* sequence obtained (808 bp) (GenBank: MH341587) was identical to that of *C. cuniculus* from a rabbit in China (GenBank: HQ397716). When samples from individual alpacas (*n* = 18) from CsVIC25 were tested individually, *C. cuniculus* VbA25 was identified in a cria of 3.6 months of age; the *gp60* sequence obtained (286 bp) (GenBank: MH356122) was 99% similar to the sequence with accession no. MG516794 from *C. cuniculus* VbA25 from a rabbit in Australia [22].

### *Giardia*

*Giardia duodenalis* was detected in six of the 81 samples tested (Table 2):

(i) *G. duodenalis* assemblage AI was detected in samples CsNSW7, CsNSW9, CsNSW21, CsSA3 and CsVIC27; the five *tpi* sequences obtained (500 bp; GenBank: MH346123) were identical to those with accession no. KM926546 and 46 other *tpi* sequences in GenBank.

(ii) *G. duodenalis* assemblage E was detected in sample CsNSW11; the sequence obtained (500 bp; GenBank: MH346124) was identical to that with accession no. GQ444456 [33] derived from a lamb in Australia.

### *Enterocytozoon*

*Enterocytozoon bieneusi* was detected in eight of the 81 samples tested (Table 2). Genotype ALP1 was detected in samples CsNSW6, CsVIC16, CsVIC22, CsSA3 and CsSA7; the five sequences obtained (243 bp; GenBank: MH342036) were identical to that with accession no. KC860942 originating from a farmed alpaca in Peru [7]. Genotype ALP3 was detected in sample CsNSW11; the one sequence obtained (243 bp; GenBank: MH342037) was identical to that with accession no. KC860930 derived from a farmed alpaca in Peru [7]. Genotype P was detected in samples CsNSW20 and CsVIC23; the two sequences obtained (243 bp; GenBank: MH342038) were identical to that with accession no. KC860928 originating from farmed alpaca in Peru [7] and accession no. AF267146 from llama (*Lama glama*) in the Munich Zoo, Germany [34].

### Epidemiological considerations

*Cryptosporidium* sp. was detected three times in pooled samples collected during the winter months; *G. duodenalis* was detected in the spring (*n* = 3), autumn (*n* = 1) and winter (*n* = 2); *E. bieneusi* was detected in the spring (*n* = 3) and winter (*n* = 5) (Table 1). None of the three pathogens was detected in the summer months. The most intensely sampled states were Victoria and New South Wales, both of which had the highest prevalences of *Cryptosporidium*, *G. duodenalis* and *E. bieneusi* (Tables 1 and 2). None of the three pathogen groups was detected in Queensland, Tasmania or Western Australia. Age data were available for most (*n* = 1313), but not all alpaca individuals. Although we did not assess the ages of all pathogen-positive individuals, as not all herd pools were examined at an individual level, all five pathogen-positive individuals were ≤ 6 months of age (Table 2). Because the cross-sectional sampling took place over a two-year period during different seasons and across different states, any epidemiological inference should be assessed with caution.

## Discussion

This is the first cross-sectional study of three eukaryotic microbes (*Cryptosporidium* sp., *G. duodenalis* and *E. bieneusi*) in alpaca herds in Australia, representing nearly 10,000 animals. In an attempt to efficiently sample herds, we elected to use a “pooling method”, which allowed us to screen animals from 81 farms across six Australian states. A total of 59 (72.9%) of the herds were included in the study, and ≥ 20% of individual herd sizes were sampled. Overall, we detected three species of *Cryptosporidium* (*C. cuniculus*, *C. parvum* and *C. ubiquitum*), two assemblages of *G. duodenalis* (AI and E) and three genotypes of *E. bieneusi* (ALP1, ALP3 and P) in 15 of the 81 herds (representing 1421 individual samples).

### Context of the molecular-genetic findings

Historically, *C. parvum* has been the most frequently recorded species of *Cryptosporidium* in molecular surveys of alpaca in the USA [35] and the UK [10, 18], and both *C. parvum* and *C. ubiquitum* have been detected in Peru [17]. However, the present study is the first report of *C. cuniculus* from alpacas. All three species of *Cryptosporidium* found in this study (*C. cuniculus*, *C. parvum* and *C. ubiquitum*) are known to be zoonotic [36], and it is proposed that alpacas acquire the infection from oocysts in their environment which originate from humans, other livestock and/or rabbits. The sequence of the subtype of *C. parvum* found (i.e. IIaA20G3R1) was a perfect match (over 785 bp) to a sequence derived from a faecal sample from a human from New South Wales [32]. This subtype is recognised as causing zoonotic infections in humans and cattle in Australia [32]. Incidentally, a study by Starkey et al. [35] used PCR to trace the zoonotic transmission of *C. parvum* from crias to six people on a farm in New York, USA. Notably, one cria that transmitted the infection to a human who did not display signs of disease (diarrhoea), indicating transmission from an alpaca with a subclinical infection [35]. The presence of *C. ubiquitum* in one of the herds in the present study could have originated from sheep, cattle or other livestock, or humans, as it is often associated with these host groups [37]. *Cryptosporidium cuniculus*, originally recorded in rabbits, has been found in humans, and there is one report from an eastern grey kangaroo [38]. The detection of *C. cuniculus* DNA in faecal samples from two alpacas from the same herd may be the result of pseudo-parasitism [39], but the possibility of it being a true infection, especially from two crias (3.6 months of age), cannot be excluded.

*Giardia duodenalis* is comprised of eight assemblages, with assemblage A being primarily associated with humans, livestock and wild ruminants, and assemblage E being common in livestock and wild ruminants [40]. Assemblages A and E have been typically reported previously in alpacas from Peru (AI, AII and E, [7, 17, 41]), the UK (A and E; [42]) and the USA (A and E; [43, 44]), and these assemblages were represented in the present study. More specifically, sub-assemblage AI was found in five of the 81 herds tested, and is considered to have zoonotic potential as it is predominantly found in humans, but has been recorded in domestic livestock and wild ruminants and occasionally in cats and dogs [40]. Aside from a mouse-derived culture of *Giardia* originating from an alpaca in Australia [45], this is the first report of *G. duodenalis* from farmed alpaca in this country.

The present study represents the first molecular investigation of *E. bieneusi* in farmed alpacas in Australia, in which eight of 81 herds were shown to be test-positive. Prior knowledge of *E. bieneusi* of alpacas is limited to one study from herds in Peru [7], and two studies in Chinese zoos [46, 47]. A Peruvian study of 126 crias discovered six novel genotypes (ALP1-6) as well as already known genotypes P, Type IV, D and Beb6 [7]. Surveys from captive alpacas kept in two Chinese zoos detected recognised genotypes J, CALTI and Beb6 [46, 47]. Additionally, a survey of captive llamas (*Lama glama*) in the Munich Zoo, Germany, identified the novel subtype P [34]. Interestingly, only alpaca- (ALP1 and ALP3) and llama-specific genotypes (P) were found in the present study. Although it is unknown whether these genotypes are zoonotic, their phylogenetic position (cf. figure 13 in [7]) suggests that they have zoonotic potential, based on epidemiological information available for other genotypes in the ‘Group 1’ clade [46].

### Prevalence

Although we were not able to calculate an overall prevalence for each of the three pathogens, given the design of this study, we were able to calculate prevalence within herds. *Cryptosporidium* sp., *G. duodenalis* and *E. bieneusi* were detected in only 3, 6 and 8 of the 81 alpaca herds, respectively (Table 1). Low herd prevalence can be attributed to a number of factors, including logistics (e.g. project design and pooling of samples), animal husbandry (e.g. herd movement, stocking density, population density during birthing, pasture management and grazing method), environmental factors (e.g. environmental temperature, humidity and rainfall) and, likely the most important factor, age.

This study indicates that young crias are more likely to harbour infection than adults (Table 2). To our knowledge, there are no examples of studies that assess *Cryptosporidium*, *Giardia* and/or *Enterocytozoon* across different age groups. Most published investigations have exclusively examined young crias (few days to several weeks of age) (cf. [16]) for the purpose of detecting pathogens associated with neonatal diarrhoea and with high morbidity [4, 9] or mortality [8, 10, 11]. The average age of the animals sampled here from each herd was 4.8 years; thus, broad sampling across ages is likely to have contributed to low prevalences. The few studies that have examined adult camelids resulted in zero prevalence of *Cryptosporidium* sp. in a herd of 53 alpaca from Japan [15] and another of 354 llamas in California, the USA [48], which examined both crias and adults. Rulofson et al. [48] also studied *G. duodenalis* and estimated a prevalence of 3% in crias, but found no infected dams. Another survey, which included adult alpacas, was that of Burton et al. [19] who examined 110 crias and their dams on 14 farms in New York and Pennsylvania, USA. The prevalence of *Cryptosporidium* was 8% in dams and 7% in crias, and 6.4% in dams and 16.3% in crias for *G. duodenalis* using direct immunofluorescence assays [19]. Two previous studies of *G. duodenalis* in 61 and 352 alpacas, respectively, showed that 3.2–26.2% of crias and 1.6–1.8% of dams were infected [41, 43]. Clearly, young crias have been reported to have a higher prevalence of *Cryptosporidium* sp. and *G. duodenalis* compared with adults, and cohabitation of crias with their dams during the time of sampling may have led to a higher prevalence in dams in some studies [48], but not in others [19]. The largest study of *Cryptosporidium* sp. in alpacas [16] examined 5163 randomly selected crias of 1 to 15 days of age from 105 herds throughout Peru and estimated a prevalence of 13% (*n* = 666). In this latter study, adults were not tested, and the testing was conducted using acid fast staining and microscopy, such that the actual prevalence could have been much higher if samples had been tested by PCR.

The study by López-Urbina et al. [16] is valuable in that it emphasises some of the important risk factors for *Cryptosporidium* sp. (which are also applicable to *G. duodenalis* and *E. bieneusi*), such as accessibility to grazing pastures and overcrowding during birthing. Stocking density was also indicated as a likely factor contributing to an outbreak of *Cryptosporidium* sp. on a farm in New York [35] and from multiple cases of *Cryptosporidium* sp. in Oregon, USA [9]. The trend toward increasing stocking densities of alpaca in the UK is also a notable risk factor for *Cryptosporidium* [18]. Additionally, a study of llamas [48] concluded that keeping animals in small pens or in large groups increases the likelihood of *G. duodenalis* infection. Larger pastures and/or the division of herds into multiple pens to achieve an acceptable stocking density might reduce the spread of other pathogens as well. The Australian Alpaca Association guidelines on herd density (stocking rate) are 10 dry sheep equivalents per hectare (DSE/ha) in areas with high rainfall, compared with 1.5 DSE/ha in drylands (www.alpaca.asn.au).

Past studies have suggested that a high pathogen prevalence is correlated with wetter seasons, as seen in the Pacific Northwest of the USA [13], and after periods of heavy rainfall in the UK [10], although other studies (cf. [9]) have found no correlation with season and that infection can occur year-round. The present investigation demonstrated that the highest prevalence of all three pathogens was in the winter season, especially when compared with summer (zero prevalence). Typically, the summers are drier than the winters in much of New South Wales and Victoria, where the vast majority of the alpaca herds were sampled in this study. The other factor is alpaca calving time, which, in Australia, is usually about two months in spring, although the timing and duration of the birthing periods can vary among farms (J. L. Vaughan, unpublished data). Crias are usually weaned at an average age of three months, when the Australian summer starts. Ultimately, longitudinal sampling of the same herds across seasons would be advantageous in future studies to understand the contribution of season and climate to pathogen prevalence.

## Conclusions

The present study provides the first baseline data set for Australia on some major eukaryotic pathogens known to affect alpaca globally. A novel host record was *C. cuniculus*, and novel locality records were made for the other pathogen species and genotypes identified. All of the pathogens characterised molecularly in this study were either known to be zoonotic or have zoonotic potential. Evident from this study was a low overall herd prevalence of *Cryptosporidium* sp., *G. duodenalis* and *E. bieneusi* infections*.* Future work should focus on pre-weaned and post-weaned crias to establish which of these pathogens play(s) a role in neonatal diarrhoea in Australia, and it would be interesting to examine faecal consistency and body condition scores to establish the clinical impact of infections by these microbes. Longitudinal studies should investigate herd densities, seasonal effects and environmental factors, such as temperature and rainfall, to ensure the health and welfare of Australia’s alpaca herds.

## Additional file

Additional file 1: **Table S1.** List of alpaca herds/farms from which faecal samples were collected for the present study. The Australian states in which samples were collected are: New South Wales (NSW), Queensland (QLD), South Australia (SA), Tasmania (TAS), Victoria (VIC), Western Australia (WA). The seasons in which herds were sampled as well as herd sizes, numbers of individuals (and %) included in pooled samples tested by PCR-based sequencing are listed. (DOCX 36 kb)

## Abbreviations

FEC: faecal egg count
*gp60*: 60-kilodalton glycoprotein
*ITS*: internal transcribed spacer
*SSU*: small subunit of nuclear ribosomal RNA gene
*tpi*: triose-phosphate isomerase
