# Supplementary material for: First cross-sectional, molecular epidemiological survey of Cryptosporidium, Giardia and Enterocytozoon in alpaca (Vicugna pacos) in Australia
Source: Parasit Vectors. 2018 Sep 5;11:498. doi: 10.1186/s13071-018-3055-6 (PMC6126005; doi:10.1186/s13071-018-3055-6)
Supplement: Supplementary file 1 — Table S1. List of alpaca herds/farms from which faecal samples were collected for the present study. The Australian states in which samples were collected are: New South Wales (NSW), Queensland (QLD), South Australia (SA), Tasmania (TAS), Victoria (VIC), Western Australia (WA). The seasons in which herds were sampled as well as herd sizes, numbers of individuals (and %) included in pooled samples tested by PCR-based sequencing are listed. (DOCX 36 kb) [file 13071_2018_3055_MOESM1_ESM.docx]

**Additional File 1**

**Table S1.** List of alpaca herds/farms from which faecal samples were collected for the present study. The Australian states in which samples were collected are: New South Wales (NSW), Queensland (QLD), South Australia (SA), Tasmania (TAS), Victoria (VIC), Western Australia (WA). The seasons in which herds were sampled as well as herd sizes, numbers of individuals (and %) included in pooled samples tested by PCR-based sequencing are listed.

| **Farm/herd/sample identification code** | **State** | **Season** | **Herd size** | **Number of individuals**  **representing the pooled sample (%)** |
| --- | --- | --- | --- | --- |
| CsNSW1 | NSW | Summer | 51 | 14 (27) |
| CsNSW2 | NSW | Spring | 35 | 16 (46) |
| CsNSW3 | NSW | Autumn | 17 | 17 (100) |
| CsNSW4 | NSW | Autumn | 21 | 21 (100) |
| CsNSW5 | NSW | Winter | 187 | 18 (10) |
| CsNSW6 | NSW | Spring | 250 | 20 (8) |
| CsNSW7 | NSW | Autumn | 59 | 18 (31) |
| CsNSW8 | NSW | Summer | 32 | 20 (63) |
| CsNSW9 | NSW | Spring | 172 | 22 (13) |
| CsNSW10 | NSW | Spring | 40 | 16(40) |
| CsNSW11 | NSW | Winter | 55 | 16 (29) |
| CsNSW12 | NSW | Winter | 70 | 15 (21) |
| CsNSW13 | NSW | Spring | 54 | 21 (39) |
| CsNSW14 | NSW | Spring | 70 | 19 (27) |
| CsNSW15 | NSW | Spring | 71 | 13 (18) |
| CsNSW16 | NSW | Summer | 70 | 20 (29) |
| CsNSW17 | NSW | Spring | 85 | 26 (31) |
| CsNSW18 | NSW | Winter | 150 | 21 (14) |
| CsNSW19 | NSW | Spring | 18 | 18 (100) |
| CsNSW20 | NSW | Spring | 17 | 7 (41) |
| CsNSW21 | NSW | Spring | 49 | 20 (41) |
| CsNSW22 | NSW | Spring | 75 | 20 (27) |
| CsNSW23 | NSW | Spring | 105 | 18 (17) |
| CsNSW24 | NSW | Winter | 17 | 17 (100) |
| CsNSW25 | NSW | Winter | 37 | 15 (41) |
| CsNSW26 | NSW | Winter | 34 | 25 (74) |
| CsQLD1 | QLD | Autumn | 100 | 23 (23) |
| CsQLD2 | QLD | Spring | 15 | 13 (87) |
| CsQLD3 | QLD | Spring | 40 | 20 (50) |
| CsQLD4 | QLD | Spring | 33 | 20 (61) |
| CsQLD5 | QLD | Winter | 55 | 10 (18) |
| CsQLD6 | QLD | Winter | 39 | 20 (51) |
| CsQLD7 | QLD | Winter | 84 | 7 (8) |
| CsSA1 | SA | Winter | 13 | 13 (100) |
| CsSA2 | SA | Summer | 20 | 17 (85) |
| CsSA3 | SA | Spring | 84 | 15 (18) |
| CsSA4 | SA | Winter | 65 | 22 (35) |
| CsSA5 | SA | Summer | 120 | 18 (15) |
| CsSA6 | SA | Winter | 150 | 22 (15) |
| CsSA7 | SA | Winter | 3000 | 10 (0.3) |
| CsTAS1 | TAS | Winter | 35 | 26 (74) |
| CsTAS2 | TAS | Winter | 57 | 26 (46) |
| CsTAS3 | TAS | Winter | 43 | 20 (47) |
| CsTAS4 | TAS | Spring | 73 | 17 (23) |
| CsVIC1 | VIC | Autumn | 18 | 15 (83) |
| CsVIC2 | VIC | Autumn | 34 | 16 (47) |
| CsVIC3 | VIC | Autumn | 117 | 17 (15) |
| CsVIC4 | VIC | Autumn | 24 | 22 (92) |
| CsVIC5 | VIC | Autumn | 33 | 17 (52) |
| CsVIC6 | VIC | Autumn | 60 | 16 (27) |
| CsVIC7 | VIC | Autumn | 150 | 22 (15) |
| CsVIC8 | VIC | Autumn | 27 | 18 (67) |
| CsVIC9 | VIC | Autumn | 70 | 19 (27) |
| CsVIC10 | VIC | Autumn | 50 | 22 (44) |
| CsVIC11 | VIC | Autumn | 48 | 18 (38) |
| CsVIC12 | VIC | Winter | 13 | 13 (100) |
| CsVIC13 | VIC | Winter | 155 | 15 (10) |
| CsVIC14 | VIC | Winter | 23 | 20 (87) |
| CsVIC15 | VIC | Winter | 30 | 13 (43) |
| CsVIC16 | VIC | Winter | 79 | 21 (27) |
| CsVIC17 | VIC | Winter | 110 | 18 (16) |
| CsVIC18 | VIC | Winter | 22 | 15 (68) |
| CsVIC19 | VIC | Winter | 37 | 20 (54) |
| CsVIC20 | VIC | Winter | 42 | 20 (48) |
| CsVIC21 | VIC | Winter | 46 | 20 (43) |
| CsVIC22 | VIC | Winter | 65 | 14 (22) |
| CsVIC23 | VIC | Winter | 175 | 25 (14) |
| CsVIC24 | VIC | Winter | 60 | 15 (25) |
| CsVIC25 | VIC | Winter | 59 | 18 (31) |
| CsVIC26 | VIC | Winter | 115 | 19 (17) |
| CsVIC27 | VIC | Winter | 13 | 12 (92) |
| CsVIC28 | VIC | Spring | 42 | 35 (83) |
| CsVIC29 | VIC | Spring | 13 | 13 (100) |
| CsVIC30 | VIC | Autumn | 75 | 15 (20) |
| CsVIC31 | VIC | Autumn | 600 | 10 (2) |
| CsVIC32 | VIC | Autumn | 780 | 10 (1) |
| CsWA1 | WA | Autumn | 68 | 24 (35) |
| CsWA2 | WA | Autumn | 650 | 14 (2) |
| CsWA3 | WA | Autumn | 96 | 14 (15) |
| CsWA4 | WA | Autumn | 15 | 9 (60) |
| CsWA5 | WA | Autumn | 25 | 5 (20) |
